# Co-expression of parathyroid hormone related protein and TGF-beta in breast cancer predicts poor survival outcome

**DOI:** 10.1186/s12885-015-1873-x

**Published:** 2015-11-23

**Authors:** Cheng Xu, Zhengyuan Wang, Rongrong Cui, Hongyu He, Xiaoyan Lin, Yuan Sheng, Hongwei Zhang

**Affiliations:** 1 Department of Breast Surgery, Yangpu Hospital, Tongji University School of Medicine, Shanghai, 200090 China; 2 Department of Intensive Care Medicine, Zhongshan Hospital, Fudan University, Shanghai, 200032 China; 3 Department of Thyroid and Breast Surgery, Changhai Hospital, Shanghai, 200433 China; 4 Department of General Surgery, Zhongshan Hospital, Fudan University, Shanghai, 200032 China

**Keywords:** Breast cancer, TGF-β, PTHrP, Prognosis, Survival analysis

## Abstract

**Background:**

Better methods to predict prognosis can play a supplementary role in administering individualized treatment for breast cancer patients. Altered expressions of PTHrP and TGF-β have been observed in various types of human cancers. The objective of the current study was to evaluate the association of PTHrP and TGF-β level with the clinicopathological features of the breast cancer patients.

**Methods:**

Immunohistochemistry was used to examine PTHrP and TGF-β protein expression in 497 cases of early breast cancer, and Kaplan-Meier method and COX’s Proportional Hazard Model were applied to the prognostic value of PTHrP and TGF-β expression.

**Results:**

Both over-expressed TGF-β and PTHrP were correlated with the tumor in larger size, higher proportion of axillary lymph node metastasis and later clinical stage. Additionally, the tumors with a high TGF-β level developed poor differentiation, and only TGF-β expression was associated with disease-free survival (DFS) of the breast cancer patients. Followed up for a median of 48 months, it was found that only the patients with negative TGF-β expression had longer DFS (*P* < 0.05, log-rank test). Nevertheless, those with higher PTHrP expression tended to show a higher rate of bone metastasis (67.6 % vs. 45.8 %, *P* = 0.019). In ER negative subgroup, those who developed PTHrP positive expression presented poor prognosis (*P* < 0.05, log-rank test). The patients with both positive TGF-β and PTHrP expression were significantly associated with the high risk of metastases. As indicated by Cox’s regression analysis, TGF-β expression and the high proportion of axillary lymph node metastasis served as significant independent predictors for breast cancer recurrence.

**Conclusions:**

TGF-β and PTHrP were confirmed to be involved in regulating the malignant progression in breast cancer, and PTHrP expression, to be associated with bone metastasis as a potential prognostic marker in ER negative breast cancer.

## Background

Breast cancer, remaining the most global common malignant tumor in women, is yearly diagnosed in more than one million cases [1]. Although the continuously improved understanding of the tumor pathology and significant advancements in diagnostic techniques have allowed more cases to be detected at an earlier stage, the overall 5-year survival rate remains low for breast cancer patients, primarily because of the high rate of recurrence and metastasis [2]. Tumor cells have been well recognized to show a distinctive attribute for metastasis to specific organs; the 1889 Stephen Paget’s original “seed and soil” hypothesis was reported that the organ-preference patterns of tumor metastasis were the product of interactions between metastatic tumor cells (the seed) and their organ microenvironment (the soil) [3]. Breast cancer is known to have a strong predilection for bone metastasis and only 20 % of the patients are still five-year alive after confirmation for the serious complication of breast cancer [4, 5].

Parathyroid hormone-related protein (PTHrP), isolated from the tumor tissues of Malignancy-associated hypercalcemia (MAH) patients, was reported to be credited for its ability to mimic parathyroid hormone (PTH) [6, 7]. During embryonic period, PTHrP plays an important role in normal mammary gland, tooth and bone development and differentiation [8–11]. PTHrP was reported to be expressed in a wide variety of fetal and adult tissues, as well as in many malignancies [6, 12]. PTHrP expression was reported to be present in many tumor types even in the absence of hypercalcemia, and related with tumor progression such as colon cancer, non-small cell lung cancer, myeloma and prostatic cancer [13–18]. In bone metastasis, PTHrP plays a key role in the osteoclastic bone resorption by stimulating receptor activator for nuclear factor-κ B ligand (RANKL) expression [19, 20]. A recent study in PyMT-MMTV breast cancer mouse model reported that PTHrP expression level was correlated with breast cancer metastasis and tumor cell survival [21].

TGF-β has been recognized to be a multi-functional growth factor involved in regulation of such processes as development, wound healing, fibrosis, carcinogenesis, angiogenesis, and immunity [22–24], and also to play a critical and double role in the progression of cancer [25, 26]. In the development of breast cancer, tumor cells obtain resistance to TGF-β-mediated growth arrest, it has been reported that TGF-β pathway retains the ability to promote the processes that support tumor progression such as tumor cell epithelial-to-mesenchymal transition, invasion, dissemination, and immune evasion [27–29]. Additionally, bone metastasis lesions-derived TGF-β can serve a critical mediator of breast carcinoma-mediated progression of osteolytic bone lesions, and the effector of this response is PTHrP. PTHrP and TGF-β can promote mutual expression and form a vicious circle [4, 19]. However, the relative quantitative expressions of TGF-β and PTHrP have not been fully explored in primary tumor tissues.

In the current study, we aimed to assess whether PTHrP and TGF-β can be dysregulated in the breast cancer tissues by analyzing clinicopathologic features and their potential value in the prognosis of breast cancer patients. The results showed that expression level of PTHrP and TGF-β in the tissues was associated with the clinicopathologic features, which could serve as an independent prognostic factor for the patients after surgery.

## Methods

### Specimen cohorts

From January 2006-December 2009, specimens were obtained from the female patients with operable primary breast cancer, who underwent treatment at the Department of Breast Surgery of Zhongshan Hospital affiliated to Fudan University and Yangpu Hospital affiliated to Tongji University School of Medicine. From a total number of the consecutive patients, we randomly selected 497 paraffin blocks of tumor tissues of the invasive patients, 341 cases from Yangpu Hospital and 156 cases from Zhongshan Hospital for the current study (random numbers table) after excluding those on neoadjuvant chemotherapy or those with positive margins on histopathology. All the patients underwent breast cancer surgery and standardized adjuvant therapies. Meanwhile, 40 specimens of benign breast tumor tissues were collected as controls. The selected patients were classified into three groups according to cTNM staging system of American Joint Committee on Cancer (AJCC), 195, 210 and 92 on stage I, II and III, respectively.

All the patients were followed up via interviews on the phone and outpatient visits every month, which began from the first postoperative day to December 2012, and ended up with 389 patients with a median of 48 months (range of 2 to 85 months), 108 patients lost during the process. After surgery, 116 patients suffered from local recurrence or distant metastasis. The local or regional recurrence was confirmed by histology and the distant metastasis was detected by biopsy or imaging techniques. By the end of this period, 26 patients had died, 21 of breast cancer, and 273 patients had developed no recurrence. The relapse-free interval (RFI) of the patients was calculated. This study was approved by medical ethics committee of Zhongshan Hospital affiliated to Fudan University (No.2010-78) and Yangpu Hospital affiliated to Tongji University (No.LL-2010-2-DOB-003) with the patient informed consents. Conforming to the principles outlined in WMA Declaration of Helsinki-Ethical Principles for Medical Research Involving Human Subjects, tissue samples were collected from Zhongshan Hospital and Yangpu Hospital at surgery, immediately fixed in formalin, and then dehydrated and embedded in paraffin.

### Immunohistochemical staining

Tissue microarrays (TMAs) were constructed containing the tumor tissues of 537 patients, 40 of whom showed benign tissues. Two core biopsies with a diameter of 0.8 mm of each case were transferred from the donor blocks to the predefined positions on the recipient paraffin blocks. The consecutive sections measured 4 μm in thickness were placed on the 3-aminopropyltriethoxysilane-coated slides.

The primary antibodies for the immunohistochemical analyses were as follows: PTHrP antibody monoclonal, (diluted 1:2000, ABGENAT), TGF-β rabbit polyclonal antibody (diluted 1:5000, SANTA CRUZ). The analyses were carried out using a two-step protocol: Upon microwave antigen retrieval, the tissues were incubated with primary antibodies overnight at 4 °C, followed by a 30-min incubation with HRP-conjugated goat anti-rabbit/mouse IgG (horseradish peroxidase-conjugated anti mouse/rabbit immunoglobin, EnVision Detection Kit A solution, Gene Tech, Hk) at room temperature, and then a 3-min incubation with diaminobenzidine, before counterstained with hematoxylin and examined under OLYMPUS BX51 microscope. Sections of human placenta were stained as PTHrP and TGF-β positive control. The negative control slides without the primary antibodies were included in all assays. All immunostained slides were reviewed and judged as positive/negative staining by two histopathologists independently in a blinded manner. In most cases, the results were identical from two pathologists, and the discrepancies were resolved by re-examination and consensus.

### Statistical analysis

The correlation of TGF-β or PTHrP expression evaluated by IHC staining and the relevant clinicopathologic features were analyzed using Pearson’s χ2 correlation test. Disease-free survival (DFS) was defined as the period from the operative date to the first recurrence (local or distant) or death of breast cancer without a recorded relapse. Cumulative survival time of each group was calculated by the Kaplan-Meier method and analyzed by the log-rank test. In the multivariate analysis, a COX’s Proportional Hazard Model was employed to estimate whether a factor was a significant independent prognostic factor of survival. All statistical tests were two-sided, and *P* values less than 0.05 were considered as statistically significant. The statistical analyses were performed using SPSS 22.0 software (SPSS Inc.).

## Results

### Immunohistochemical tissue staining

**Fig. 1:**
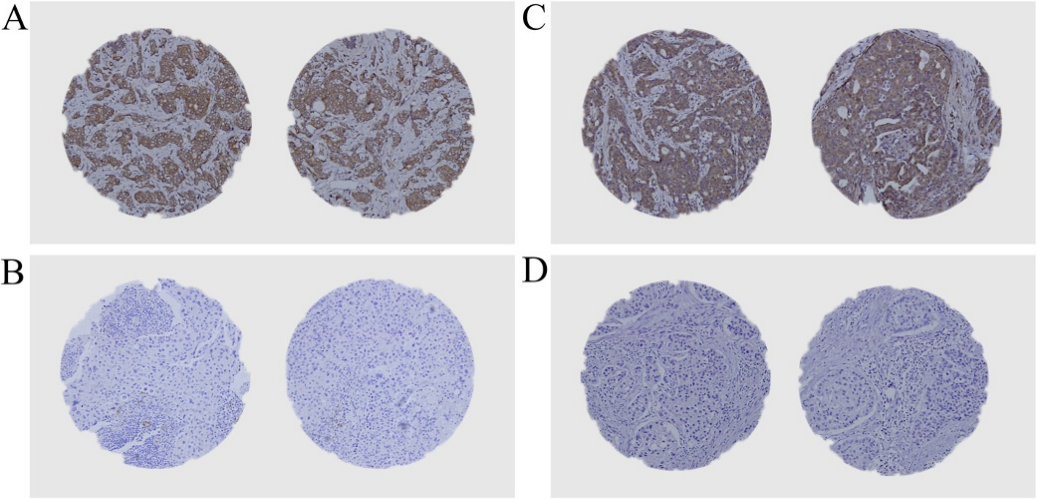
Photographs of TGF-β and PTHrP expression in breast cancer tissues by immunohistochemical staining. **a**.**b** Representative images of TGF-β positive (**a**) or TGF-β negative (**b**) cases with immunostaining (magnification × 200); **c**.**d** Representative images of PTHrP positive (**c**) or PTHrP negative (**d**) cases with immunostaining (magnification × 200)

An analysis was made of a tissue obtainment containing 497 breast cancer patients and 40 benign breast tumor patients, all females, with the median age of 56.7 in the former (ranging from 26 to 95), and with the median age 43.2 in the latter (from 22 to 72). The staining of TGF-β and PTHrP was mainly observed on the cytoplasm of cells in the breast tumor tissues (Fig. 1a, c), and most of the stroma areas were negative staining. Almost half of the breast tumors exhibited positive levels of TGF-β and PTHrP expression, 55.1 % in 274 cases, 54.5 % in 271 cases, respectively. Both TGF-β and PTHrP positive staining were rarely detected in the benign breast tissues, 10 % in 4 cases and 17.5 % in 7 cases, respectively.

### Correlation of TGF-β expression and clinicopathologic features

All breast cancer cases were separated into two groups as TGF-β positive and TGF-β negative based on the TGF-β staining degree of the tumor sections. Compared with those with TGF-β negative staining, the patients with TGF-β positive had poor differentiation in histology and larger tumor size, and most in the positive group showed a higher proportion of axillary lymph node metastasis and later clinical stages. In the two groups, however, no significant difference was observed on patients’ age, skin involvement, pathological type, and expression of estrogen receptor and HER-2 (Table 1).

**Table 1:** Clinicopathologic features and TGF-β expression

	TGF-β positive No. (%)	TGF-β negative No. (%)	χ^2^	*P* value
Age				
≤55	156 (56.9 %)	111 (49.8 %)	2.534	0.111
>55	118 (43.1 %)	112 (50.2 %)		
Tumor size				
≤2 cm	137 (50.0 %)	141 (63.2 %)	8.729	0.003
>2 cm	137 (50.0 %)	82 (36.8 %)		
Skin involvement^a^				
No	229 (83.6 %)	190 (85.2 %)	0.245	0.620
Yes	45 (16.4 %)	33 (14.8 %)		
LN metastasis				
No	154 (57.5 %)	148 (67.9 %)	5.556	0.018
Yes	114 (42.5 %)	70 (32.1 %)		
Unknown	6	5		
Histologic grade				
≤II	182 (67.7 %)	169 (78.2 %)	6.710	0.01
>II	87 (32.3 %)	47 (21.8 %)		
Unknown	5	7		
Clinical stage				
I	91 (33.2 %)	104 (46.6 %)	9.329	0.009
II	128 (46.7 %)	82 (36.8 %)		
III	55 (20.1 %)	37 (16.6 %)		
ER				
(−)	75 (28.7 %)	59 (27.6 %)	0.079	0.779
(+)	186 (71.3 %)	155 (72.4 %)		
Unknown	13	9		
HER-2				
(−)	94 (38.7 %)	83 (41.7 %)	0.417	0.518
(+)	149 (61.3 %)	116 (58.3 %)		
Unknown	31	24		
Tumor type				
IDC^b^	234 (85.4 %)	189 (84.8 %)	0.041	0.840
Non-IDC^c^	40 (14.6 %)	34 (15.2 %)		

^a^skin involvement: edema, redness, nodularity, or ulceration

^b^IDC, invasive ductal carcinoma

^c^Non-IDC: invasive lobular carcinoma, mucinous or colloid carcinoma, medullary carcinoma, metaplastic carcinoma

### Correlation of PTHrP expression and clinicopathologic features

The histopathological parameters were further compared in both PTHrP positive and negative group. Analogously, the results showed that in the positive group, larger tumor size, higher proportion of axillary lymph node metastasis and later clinical stages were observed, and that the two groups displayed no significant distinction in patients’ age, skin involvement, degrees of pathological differentiation, pathologic type of cancer, level of estrogen receptor (ER) and HER-2 (Table 2).

**Table 2:** Clinicopathologic features and PTHrP expression

	PTHrP positive	PTHrP negative	χ^2^	*P* value
	No. (%)	No. (%)		
Age				
≤55	146 (53.9 %)	121 (53.5 %)	0.006	0.941
>55	125 (46.1 %)	105 (46.5 %)		
Tumor size				
≤2 cm	133 (49.1 %)	145 (64.2 %)	11.372	0.001
>2 cm	138 (50.9 %)	81 (35.8 %)		
Skin involvement^a^				
No	223 (82.3 %)	196 (86.7 %)	1.834	0.176
Yes	48 (17.7 %)	30 (13.3 %)		
LN metastasis				
No	154 (57.7 %)	148 (67.6 %)	5.014	0.025
Yes	113 (42.3 %)	71 (32.4 %)		
Unknown	4	7		
Histologic grade				
≤II	187 (70.8 %)	165 (74.7 %)	0.885	0.347
>II	77 (29.2 %)	56 (25.3 %)		
Unknown	7	5		
Clinical stage				
I	95 (35.1 %)	100 (44.2 %)	6.937	0.031
II	116 (42.8 %)	94 (41.6 %)		
III	60 (22.1 %)	32 (14.2 %)		
ER				
(−)	78 (29.2 %)	56 (26.9 %)	0.303	0.582
(+)	189 (70.8 %)	152 (73.1 %)		
Unknown	4	18		
HER-2				
(−)	98 (40.3 %)	79 (39.7 %)	0.018	0.893
(+)	145 (59.7 %)	120 (60.3 %)		
Unknown	28	27		
Tumor type				
IDC^b^	231 (85.2 %)	192 (85.0 %)	0.008	0.929
Non-IDC^c^	40 (14.8 %)	34 (15.0 %)		
TGF-β				
(−)	116 (42.8 %)	107 (47.3 %)	1.027	0.311
(+)	155 (57.2 %)	119 (52.7 %)		

^a^skin involvement: edema, redness, nodularity, or ulceration

^b^IDC, invasive ductal carcinoma

^c^Non-IDC: invasive lobular carcinoma, mucinous or colloid carcinoma, medullary carcinoma, metaplastic carcinoma

The previously reported results suggested that the high levels of TGF-β and PTHrP were significantly correlated with the features of more advanced breast cancer such as larger tumor size, higher proportion of axillary lymph node metastasis and later clinical stages. In the current study, however, such features showed little association with these two oncoprotein levels. Remarkably, only breast cancer patients with high level of TGF-β presented poor pathological differentiation.

### TGF-β/PTHrP expression and survival

**Fig. 2:**
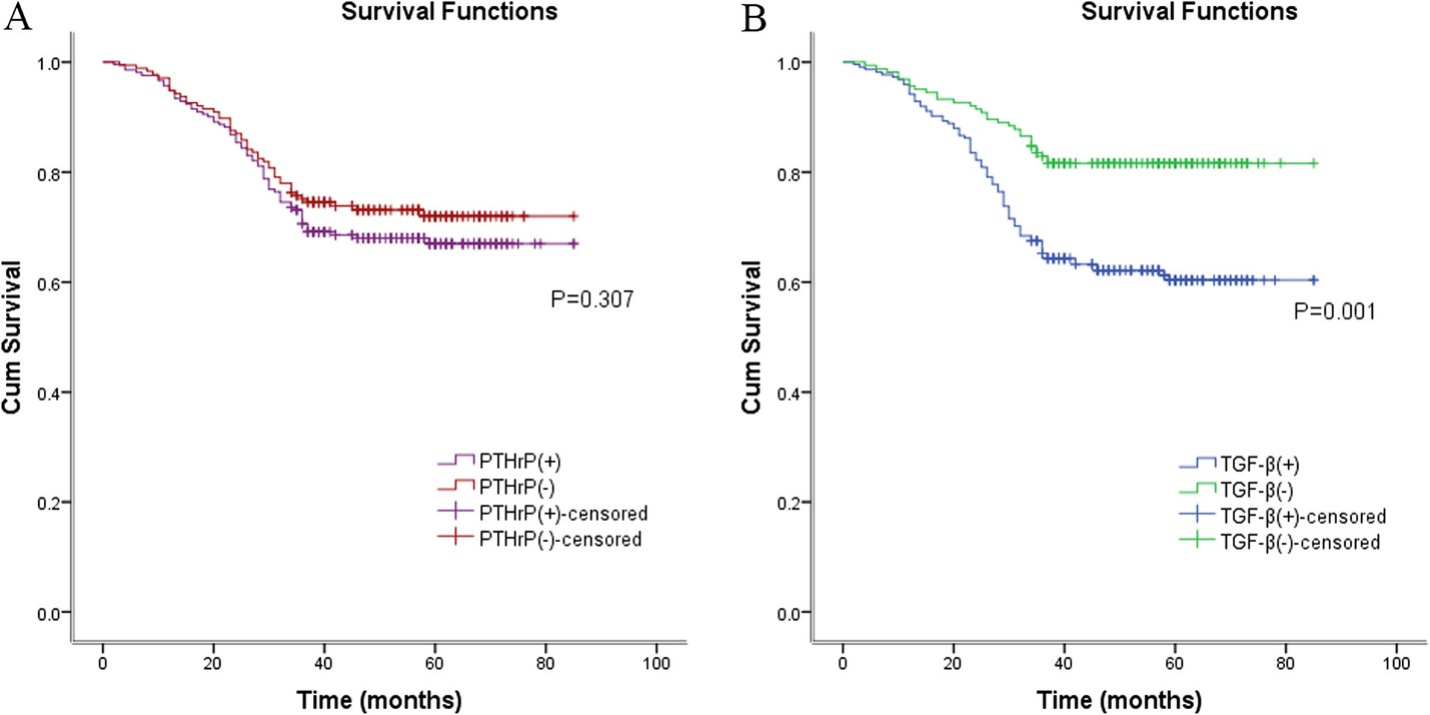
**a**. Kaplan–Meier analyses of the effect PTHrP expression on DFS (*P* = 0.307, log-rank test); **b**. Kaplan-Meier analyses of the effect TGF-β expression on DFS (*P* = 0.001, log-rank test)

As indicated by the findings of the 389 follow-ups, the recurrence rate was approximately 32.1 % and 27.1 % in 212 and 177 cases in PTHrP positive and negative group, respectively. Those who displayed higher PTHrP expressions tended to have a higher bone metastasis rate (67.6 % vs. 45.8 %, *P* = 0.019) (Table 3), but the data of Kaplan-Meier lifetime analysis showed no significant difference of the cumulative DFS in the positive and negative group, respectively (Fig. 2a).

**Table 3:** Recurrence or metastasis in PTHrP positive/negative expression in breast cancer patients

	Site of recurrence	PTHrP positive	PTHrP negative	Statistical values	*P* value
		No. (%)	No. (%)		
local recurrence	Chest wall or regional LN	3 (4.4 %)	5 (10.4 %)	Fisher	0.272
metastasis	bone	46 (67.6 %)	22 (45.8 %)	χ^2^ = 5.52	0.019
	lung	12 (17.6 %)	13 (27.1 %)		
	liver	6 (8.82 %)	8 (16.7 %)		
	brain	1 (1.47 %)	0 (0.0 %)		
No. of recurrence		68 (32.1 %)	48 (27.1 %)	χ^2^ = 1.133	0.287
No. of no recurrence		144 (67.9 %)	129 (72.9 %)

**Fig. 3:**
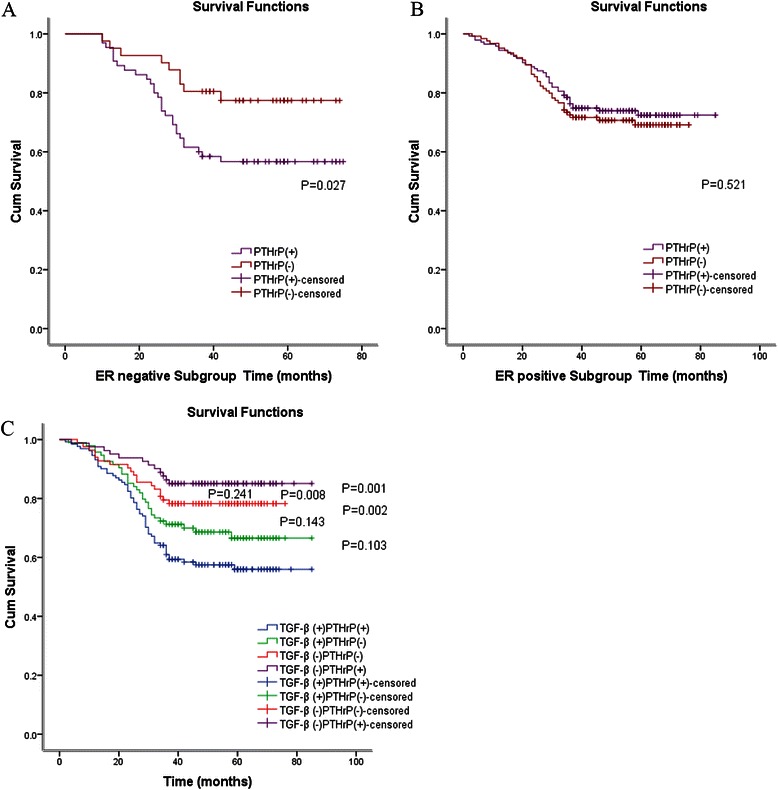
**a**. Kaplan-Meier analyses of the effect PTHrP expression in ER negative subgroup on DFS (P=0.027, log-rank test) **b**. Kaplan-Meier analyses of the effect PTHrP expression in ER positive subgroup on DFS (*P* = 0.521, log-rank test) **c**. Kaplan-Meier analyses of the effect PTHrP and TGF-β expression on DFS

To examine further the relationship between TGF-β level and breast cancer patients’ survival, all cases were divided into two groups based on TGF-β level; consequently, there were 225 cases of TGF-β positive. As shown by Fig. 3, when compared with high TGF-β controls, the group without detectable TGF-β expression was significantly associated with longer DFS among 164 patients (*P* < 0.05, log-rank test) (Fig. 2b).

When all cases were further divided into two subgroups by the expression of estrogen receptor staining signal, Kaplan-Meier lifetime analysis demonstrated that those who expressed lower PTHrP in ER negative subgroup had favorable prognosis (*P* < 0.05, log-rank test) (Fig. 3a). In ER positive group, nevertheless, no statistically significant differences were observed in the prognosis of those who had positive or negative PTHrP expression (3b). Contrastive analysis of the DFS in ER and PTHrP groups proved that those with negative ER and positive PTHrP expression developed the worst prognosis.

As indicated by DFS curves constructed for the comparison of four different groups based on PTHrP and TGF-β survival results, the prognosis of those with positive TGF-β and PTHrP expression was obviously worse than that of those with negative TGF-β, independently of PTHrP changes (Fig. 3c). These results clearly indicated a statistically significant correlation between PTHrP/TGF-β up-regulation and poorer survival outcome.

Based on the results from the multivariate COX’s Proportional Hazard Model to evaluate the clinical values of TGF-β/PTHrP in prognosis, it was found that the abnormal expression of TGF-β was an independent prognostic factor for DFS in breast cancer patients (HR = 0.469, 95.0 % CI 0.301 to 0.729; *P* < 0.05). The results also revealed that proportion of axillary lymph node metastasis and histologic grade were related to the prognosis of breast cancer. More importantly, the high proportion of axillary lymph node metastasis was the most effective unfavorable prognostic factor in DFS (HR = 2.054, 95.0 % CI 1.398 to 3.019; *P* < 0.05). However, the multivariate analysis indicated that PTHrP was not an independent prognostic factor in breast cancer (HR = 1.022, 95.0 % CI 0.684 to 1.527; *P* > 0.05) (Table 4).

**Table 4:** Multivariate analyses of DFS (Backward Stepwise, Likelihood Ratio)

Variable	HR	95 % CI	*P* value
Tumor size	0.954	0.636 to 1.432	0.821
(≤2 cm vs >2 cm)			
Skin involvement			
(No vs Yes)	1.211	0.752 to 1.950	0.431
LN metastasis	2.054	1.398 to 3.019	0.001
(No vs Yes)			
Histologic grade	1.553	1.050 to 2.298	0.028
(≤II vs > II)			
ER	0.747	0.496 to 1.125	0.163
(negative vs positive)			
TGF-β			
(positive vs negative)	0.469	0.301 to 0.729	0.001
PTHRP	1.022	0.684 to 1.527	0.916
(positive vs negative)			

## Discussion

Breast cancer originates in mammary epithelial cells, with a clear tendency to lymph node and blood metastasis; however, PTHrP is expressed in the normal epithelial cells, but its expression rises in breast cancer, becoming associated with multiple metastatic lesions. Recently, Ghoussaini et al. combined several datasets, encompassing 70,000 patients and 68,000 controls and identified rs10771399, a 300 kb linkage disequilibrium block that contains only one gene, PTHrP, one of the candidate genes connecting with the mammary gland development and breast cancer bone metastasis [30]. Li J et al. used the MMTV-Cre transgene to target the PTHrP gene in mammary epithelial cells in PyMT-MMTV GEM model, finding out that it could prolong tumor latency, inhibit tumor growth and repress metastases. Tumor growth inhibition was reported to be correlated with reduced proliferation and increased apoptosis [21]. But according to the previously reported clinical researches, the relation between PTHrP and tumor progression remains controversial. As reported by Linforth R, PTHrP was expressed in 68 % of surgically excised early breast cancers, when compared with 100 % bone metastases; and co-expression of both PTHrP and receptor predicted the worst clinical outcome [31]. However, another investigation of 3 year-postoperative following-ups found no difference in PTHrP expression of the primary tumor amongst the metastasis-free group, distant-recurrence group and other preoperative distant disease group [32]. The results reported by Surowiak showed that the patients with high expression of PTHrP manifested longer survival than those with lower PTHrP expression [33]. Additionally, a retrospective clinical study of breast tumors collected at surgery suggested better outcome and survival in the patients whose primary tumor over-expressed PTHrP [34]. The current study was an assessment of the inconsistent results derived from the previously reported clinical researches.

The important role of TGF-β in breast cancer development has been extensively investigated. Recent studies have revealed that TGF-β activates its receptors through ligand binding, thus resulting in a further activation of Smad family proteins through phosphorylation, and that nuclear-localizated Smad proteins regulates the transcription of target genes [35]. TGF-β, a potent mediator of growth inhibition, is capable of inducing apoptosis in a variety of tumors at early stage. In the advanced tumors, however, TGF-β activation seems to enhance breast tumor growth and invasion. As a critical negative regulator of the immune system, TGF-β inhibits T cells and antigen present cell by preventing cell-mediated tumor clearance in tumor progression [36, 37]. TGF-β functions as a potent inducer of breast cancer angiogenesis by increasing the expression of vascular endothelial growth factor (VEGF) expression [38, 39]. It has been suggested that TGF-β can cause epithelial-mesenchymal transition (EMT) via Smad pathway and its downstream effect genes, and also up-regulate plasminogen activator, MMP-2 and MMP-9, which degrade extracellular matrix, allowing for subsequent migration of breast cancer cells [40–42]. As a possible trigger of breast cancer metastasis, additionally, TGF-β which regulates PTHrP is simultaneously stimulated by PTHrP in bone metastasis [43, 44]. Therefore, the approach we developed in the current study could be a co-detection of the progression and prognosis based on these gene expressions in breast cancer tissues.

In the current study, we further investigated the correlation between PTHrP/TGF-β expression and clinicopathologic features of breast cancer. We found that positive expression of PTHrP/TGF-β was linked to larger tumor size, higher proportion of axillary lymph node metastasis and later clinical stages. The cancer biological functions of PTHrP were reported as followings: PTHrP can promote primary tumor proliferation by activing PI3K-Akt, AKR1C3 pathway and affect cell cycle progression by up-regulating Cyclin D2 and Cyclin A2 protein levels [17, 45–48]; PTHrP can play an autocrine neoplastic role in evading apoptosis by decreasing the levels of Beclin1 and LC3-II or controlling the Bcl-2 and Caspase family [45, 49, 50]; PTHrP accelerates the adhesion, invasion and metastasis of tumor cells to the bone [51, 52]; and local increased PTHrP secreted by the breast cancer metastatic sites stimulating osteoblasts to express RANKL and inhibit osteoprotegerin (OPG) secretion, PTHrP has been shown to play a key role in the osteolytic resorption of bone metastasis by activating osteoclast division and growth [53]. PTHrP expression has been shown to be under the control of numerous growth and angiogenic factors such as TGF-β, VEGF, epidermal growth factor (EGF) and platelet-derived growth factor (PDGF), and meanwhile it stimulates the expression of these factors in various cell types and behaves as an angiogenic factor in endothelial cells [54]. Combined these protein functions with our research data, we consider both TGF-β and PTHrP as oncogenes in breast cancer.

It followed that either PTHrP-positive or TGF-β-positive breast cancer patients indicated a high risk of metastasis. Bone, followed by the lung and liver, is one of the most preferential metastatic target sites for breast cancer [2]. It has been well recognized that breast cancer cells spread to distant target organs with their own inherent character. Our research suggested that the higher PTHrP expression the patients tended to have, the higher bone metastasis rate would be (67.6 % vs. 45.8 %, *P* = 0.019). Furthermore, the results of the cross analysis between different groups showed that the prognosis of the patients with both positive TGF-β and PTHrP expression was apparently worse than all the others. According to the Stanley Paget “seed and soil” hypothesis, tumor cells as “seed” invading bone provide additional growth factors that activate the bone microenvironment as “soil,” which in turn produces growth factors that feed the tumor cells, creating a vicious cycle of destructive mutual cooperation [55]. Combined with our current results, the breast cancer cells which expressed both TGF-β and PTHrP can be the competent seed that has the capacity to metastasize to bone. As indicated by the survival analysis, though no significant difference was observed in the cumulative DFS of 212 cases with PTHrP expression and 177 cases with PTHrP negative, the findings were consistent with those previously reported. In ER negative subgroup, however, those who expressed positive PTHrP expression presented poor prognosis, the findings consistent with the results found in genetically engineered PyMT-MMTV GEM model which is not representative of ER positive breast cancer [56]. Usually, ER positive breast cancer accounts for 60 to 70 % of all breast cancers. In our study, we can find out that the case number and overall DFS of ER positive subgroup was superior to ER negative subgroup. We suppose that this is the reason why there was no significant difference of the cumulative DFS in the PTHrP positive and negative group.

Metastasis and recurrence of breast cancer postoperatively is probably the major reason of treatment failure or even death. Further studies on the prognostic factors of recurrence and metastasis are essential to breast cancer treatment. In the current study, Cox regression analysis was applied to determining significant prognostic factors, the results of which showed that TGF-β expression, LN metastasis and histologic grade can be the significant prognostic factors. The patients with LN metastasis were found to be more likely to relapse, the hazard ratio of DFS is 2.054 (*P* < 0.01), indicating that such patients may have about 2 times more risk of breast cancer relapse; and the hazard ratio of DFS for TGF-β is 0.469 (*P* < 0.01), indicating that those with negative TGF-β might reduce the relapse risk by about 53.1 %. However, PTHrP expression was neither a new independent prognostic factor nor a single therapeutic target in breast cancer (HR = 1.022; 95.0 % CI 0.684 to 1.527; *P* > 0.05). Although it was related to the cancer development process, PTHrP expression was even of some survival advantage in the subgroup of the patients with ER positive breast cancer. A recent experiment suggested that curcuminoids inhibited TGF-β-induced PTHrP by decreasing phospho-Smad2/3 and Ets-1 protein levels, thus reducing osteolytic bone destruction [57]. In conclusion, TGF-β and PTHrP mediated double-targeted therapy can be well considered as a novel treatment in breast cancer.

Tumor occurrence and development can be considered as the accumulation of gene mutations and epigenetic modifications. The predominant consequence of this accumulation is the activation of proto-oncogenes or silencing of tumor-suppressor genes [58]. Consistent with previous reports that PTHrP can promote the occurrence or development of malignant tumors through various mechanisms, our results suggested the advanced extent of breast cancer was correlated with TGF-β and PTHrP co-expression. More importantly, the patients with both positive TGF-β and PTHrP expression were significantly associated with poorest DFS, and the patients with positive PTHrP expression had worse cumulative survival in ER negative breast cancer. These results together indicated that TGF-β and PTHrP co-expression could act as proto-oncogenes in the development of breast cancer and that double-targeted therapy could be considered as a novel therapy for breast cancer.

## Conclusions

As verified by the current study, co-expression of TGF-β and PTHrP can be associated with breast cancer progression, recurrence and poor postoperative survival outcomes. PTHrP expression in breast tumors is relevant to bone metastasis. PTHrP expression can act as a potential prognostic tool in ER negative breast cancer.

## Abbreviations

AJCC: American Joint Committee on Cancer
DFS: disease-free survival
EGF: epidermal growth factor
EMT: epithelial-mesenchymal transition
ER: estrogen receptor
MAH: malignancy-associated hypercalcemia
OPG: osteoprotegerin
PDGF: platelet-derived growth factor
PTH: parathyroid hormone
PTHrP: parathyroid hormone related protein
RANKL: receptor activator for nuclear factor-κ B ligand
RFI: relapse-free interval
TMAs: tissue microarrays
VEGF: vascular endothelial growth factor
